# Modeling Reveals the Role of Aging and Glucose Uptake Impairment in L1A1 *Listeria monocytogenes* Biofilm Life Cycle

**DOI:** 10.3389/fmicb.2017.02118

**Published:** 2017-11-01

**Authors:** Eva Balsa-Canto, Carlos Vilas, Alejandro López-Núñez, Maruxa Mosquera-Fernández, Romain Briandet, Marta L. Cabo, Carlos Vázquez

**Affiliations:** ^1^(Bio)Process Engineering Group, IIM-CSIC Spanish National Research Council, Vigo, Spain; ^2^Mathematics Department, ITMATI, CITIC, University of A Coruña, A Coruña, Spain; ^3^Microbiology Group, IIM-CSIC Spanish National Research Council, Vigo, Spain; ^4^Micalis Institute, INRA, AgroParisTech, Université Paris-Saclay, Massy, France

**Keywords:** *L. monocytogenes*, biofilm, dynamic modeling, parameter estimation, biofilm aging, glucose impaired uptake

## Abstract

*Listeria monocytogenes* is a food-borne pathogen that can persist in food processing plants by forming biofilms on abiotic surfaces. The benefits that bacteria can gain from living in a biofilm, i.e., protection from environmental factors and tolerance to biocides, have been linked to the biofilm structure. Different *L. monocytogenes* strains build biofilms with diverse structures, and the underlying mechanisms for that diversity are not yet fully known. This work combines quantitative image analysis, cell counts, nutrient uptake data and mathematical modeling to provide a mechanistic insight into the dynamics of the structure of biofilms formed by *L. monocytogenes* L1A1 (serotype 1/2a) strain. Confocal laser scanning microscopy (CLSM) and quantitative image analysis were used to characterize the structure of L1A1 biofilms throughout time. L1A1 forms flat, thick structures; damaged or dead cells start appearing early in deep layers of the biofilm and rapidly and massively loss biomass after 4 days. We proposed several reaction-diffusion models to explain the system dynamics. Model candidates describe biomass and nutrients evolution including mechanisms of growth and cell spreading, nutrients diffusion and uptake and biofilm decay. Data fitting was used to estimate unknown model parameters and to choose the most appropriate candidate model. Remarkably, standard reaction-diffusion models could not describe the biofilm dynamics. The selected model reveals that biofilm aging and glucose impaired uptake play a critical role in L1A1 *L. monocytogenes* biofilm life cycle.

## 1. Introduction

*Listeria monocytogenes* is a Gram-positive, food-borne pathogen that can cause systemic infections in immune compromised, pregnant or elder patients (Cossart and Lebreton, 2014). Thirteen serotypes of *L. monocytogenes* have been reported from which three −1/2a, 1/2b, and 4b– account for the majority of human disease (Swaminathan and Gerner-Smidt, 2007). The primary mode of transmission of this pathogen to humans is the consumption of contaminated food (Kathariou, 2002; Swaminathan and Gerner-Smidt, 2007). Food gets contaminated by contact with unhygienic work surfaces and facilities where biofilms are found (Wilks et al., 2006).

Biofilms are microbial communities that appear in biotic or abiotic surfaces (Costerton et al., 1987). *L. monocytogenes* can form biofilms on common food contact surfaces, such as plastic, polypropylene, rubber, stainless steel, and glass (Silva et al., 2008). In biofilms, *L. monocytogenes* finds protection from a variety of environmental factors, such as temperature, sugar, salt or pH (Møretrø and Langsrud, 2004) and tolerates better biocides, hampering the process of decontaminating surfaces (Carpentier and Cerf, 2011). In fact, many *L. monocytogenes* strains have been isolated from food processing plants (Rodríguez-López et al., 2015) despite the programs to sanitize industrial facilities.

Resistance to stress, including resistance to biocides, is largely associated with biofilm structure (Costerton et al., 1987; Donlan and Costerton, 2002). Most biofilms exhibit complex structures in that patches of cell aggregates are scattered throughout an exopolysaccharide matrix, creating channels.

Plate counts have been widely used to analyze biofilms. However, plate counts may be misleading in the study of the progress of biofilms (Daims and Wagner, 2007), as they include only viable culturable cells and do not inform about the structure.

Imaging techniques allow for a more comprehensive study of biofilms. They allow mapping viable and damaged or dead cells (Tawakoli et al., 2013) or the distribution of extracellular polymeric substance but also reconstructing three-dimensional structures. Quantitative image analysis provides further insights by computing parameters which characterize structures of biofilms (Yang et al., 2000).

Recent works suggest several alternative work-flows and software tools for the systematic analysis of microscopy images. IMARIS (commercial software) enables the reconstruction of 3D structures. COMSTAT (Heydorn et al., 2000), ISA 3D (Beyenal et al., 2004), or PHLIP (Mueller et al., 2006) allow quantifying confocal laser scanning microscopy (CLSM) images. BIOFILMDIVER (Mosquera-Fernández et al., 2014) permits the quantification of 2D images for epifluorescence and CLSM. Vyas et al. (2016) used machine learning algorithms to analyse scanning electron microscopy images.

Modeling is a complementary tool for studying biofilm dynamics. The emphasis is paid into qualitative validations of developed models, which are able to recover experimentally observed structures. In that pursue, the new generation of biofilm models offer detailed descriptions of the formation of heterogeneous structures with clusters and mushrooms (see the reviews by Picioreanu et al., 2004; Wanner et al., 2006; Horn and Lackner, 2014). Continuous and hybrid models have been proposed that incorporate various mechanisms to describe biomass growth, spreading and detachment as well as nutrients transport and conversion.

Biofilms of *L. monocytogenes* show a variety of structures: mono-layers of adhered cells, flat unstructured multi-layers, honeycomb structures or clusters (Chae and Schraft, 2000; Djordjevic et al., 2002; Marsh et al., 2003; Rieu et al., 2008; Bridier et al., 2010; Pilchová et al., 2014). Recently, Guilbaud et al. (2015) reconstructed CLSM images to observe *L. monocytogenes* intra-species diversity in forming biofilms. The work considers the biofilm structures formed by 96 isolates and concludes that most strains form complex honeycomb-like structures at 48 h.

Previous works (Mosquera-Fernández et al., 2014, 2016) used quantitative image analysis throughout time to study the life cycle of biofilms formed by three *L. monocytogenes* strains. The analysis showed the presence of, at least, three phases: separate clusters which evolve to interconnected clusters, honeycomb-like or flat structures and a final detachment. The rates at which these phases occur vary significantly among strains.

In this study we focus our attention in the biofilms formed by L1A1 strain (serotype 1/2a–3a, lineage II). L1A1 biofilms exhibited a clearly distinctive dynamics: a fast transition toward a flat biofilm, which increases in thickness for a couple of days to massively detach after 4 days (Mosquera-Fernández et al., 2016). The mechanisms that explain this characteristic dynamics are not yet fully known.

The aim of this work is to provide some novel insights into the mechanisms that drive the dynamics of the biofilms formed by L1A1 *L. monocytogenes* strain. For this purpose, we combined quantitative image analysis, cell counts, nutrient uptake tests and modeling through a data-based model identification loop. We selected a deterministic continuous modeling framework. Deterministic reaction-diffusion models (RDM) offer the advantage of the reproducibility (Wanner et al., 2006). Besides, this type of models can be solved with advanced numerical techniques to guarantee the computational efficiency required for model identification through optimization based techniques (Balsa-Canto et al., 2010; Vilas et al., 2017). We formulated various candidate reaction-diffusion models to describe the system. The candidate models incorporate alternative mechanisms for growth, nutrient consumption, and detachment. Each model was reconciled with the measured data through optimization based data fitting. The best model was selected attending to a best compromise between the number of unknown parameters and its capability to quantitatively reproduce the measurements.

## 2. Materials and methods

### 2.1. Experimental methods

#### Bacterial culture conditions

Bacteria tested was *L. monocytogenes* L1A1 (serotype 1/2a/3a, lineage II) isolated from thermal gloves used in the fishing industry by the Microbiology and Marine Technology Products research group at IIM-CSIC.

Bacterial stock cultures were kept at −80°C in tryptone soy broth, TSB (BD Difco, USA), containing 50% glycerol in the ratio 1:1(υ/υ). For each experiment, L1A1 cells were grown in consecutive subcultures in TSB medium for 8 and 16 h at 37°*C* (optimal growth temperature). The subcultures were adjusted to an *OD*700 nm of 0.1(±0.001) which corresponds to approximately 10^8^ CFU/ml.

#### Biofilm formation

A volume of 50 μl of the overnight subculture was added to 200 μl of fresh TSB in each well of polystyrene of a 96-well microtiter plate (Greiner Bio-one, France). Microplates were incubated at 25°C under static conditions.

After 1 h of adhesion, the wells were rinsed to eliminate any non-adherent bacteria before being refilled with 200 μl of fresh medium. Biofilm harvesting was performed eight times per strain at 1, 4, 24, and every 24 h up to 120 h.

#### Image acquisition by confocal laser scanning microscopy (CLSM)

Images acquisition was performed using the high throughput method described by Bridier et al. (2010). The method relies on the use of microtiter plates with a μ-clear base allowing for a high-resolution imaging of biofilms. For visualization, we used the FilmeTracer LIVE/DEAD Biofilm Viability kit (Invitrogen, USA). The kit contains two different fluorescent nucleic acid markers, SYTO and propidium iodide (PI). SYTO fluorochrome penetrates in all cells while PI only penetrates in damaged cells. The fluorochromes generate a bi-color labeling in such a way that viable cells appear in green and non-viable cells in red.

A Leica SP2 AOBS confocal laser scanner microscope (Leica Microsystems, France) was used at the INRA MIMA2 Imaging platform. Scans were obtained at 400 Hz using 63 × 0.8 NA oil immersion objective with a 488 nm argon laser set at 25% of intensity.

One horizontal cross-section of biofilm, corresponding to a real biofilm area of 238 × 238 μm^2^, defines one image or slice. Horizontal cross-sections were acquired consecutively along z-axis using a scanning step size of 1 μm. The collection of horizontal cross-sections corresponds to a stack. We collected seven stacks at diverse randomly selected locations in the biofilms. Two biofilm replicas per sampling time were considered (a total of 7 × 2 × 7 stacks). For each image acquisition, we recorded dual (green and red) emissions.

#### Image visualization

We used IMARIS software (www.bitplane.com/imaris/imaris) for the visual inspection of the images at different times and heights. IMARIS offers several view modes. The slice 2D mode allows the visualization of one horizontal cross-section at a particular height, i.e., at a given slice of the stack. The blend 3D view mode provides an aerial view of the observed region, thus helping to identify biofilm structures. The section view mode lets the user inspect several slices together. Moreover, the gallery view mode displays all slices as a continuous series of images allowing the user to analyze how the structure evolves along the z-axis. Green and red channels—GCh, green channel, corresponding to live cells and RCh, red channel, to non-viable cells—can be visualized separately allowing to easily detect the appearance of non-viable cells during the biofilm life cycle.

#### Number of adherent cells

The number of adherent cells was determined according to Herrera et al. (2007). Samples were collected at 1 and 96 h from squares removed from the microtiter cavities and immersed in 10 ml of phosphate-buffered saline for 10 s to release non-adherent cells. Adherent cells were collected with peptone water-moistened swabs. After the squares had been rubbed twice with the swabs, they were transferred to 10 ml peptone water and subjected to 1 min of vortexing. The number of adherent cells was determined by plating the appropriated serial dilutions on tryptic soy agar (Cultimed, Spain) after incubation at 37°C for 24 h.

#### Analytical methods

At each sampling interval, the bulk was poured to an eppendorf and centrifuged at 9,000 g during 10 min. Supernatant was used to determine the carbon and nitrogen sources, sugar (Bernfeld, 1951) and protein (Lowry et al., 1951) contents for nutrient analysis. Sediment was washed twice with distilled water and dried to constant weight at 106°C for biomass estimation.

### 2.2. Theoretical methods

#### Image quantitative analysis

We used IMARIS software to compute the maximum thickness (MxT) of formed biofilms. MxT values were extracted for the eight replicas at each sampling time (up to 120 h) when cells were organized forming a clearly differentiable biofilm. The mean values obtained out of the eight replicas were used as the basis for model identification.

BIOFILMDIVER (Mosquera-Fernández et al., 2014) was used to obtain the area covered by cells (CA) for each slice. CA corresponds to the ratio between the number of colored pixels and the total number of pixels. In this way, CA = 0% is equivalent to the absence of cells while CA ~100% corresponds to a densely occupied slice. The CA value was computed for the biofilm including both channels (GCh and RCh) but also for both channels separately to analyse when and where non-viable cells appear in the biofilm.

#### Modeling approach

Model building was formulated as an iterative approach. To elucidate the mechanisms explaining the life cycle of the biofilms formed by L1A1 *L. monocytogenes* strain we proposed a set of candidate models M1–M4. Each model incorporated different mechanisms which in turn called for various unknown parameters. Unknown parameters were estimated using data fitting techniques within the AMIGO2 toolbox (Balsa-Canto et al., 2016).

For the sake of computational efficiency and reproducibility, all candidate models correspond to deterministic reaction-diffusion models in one dimension. The models consist of a set of (non-linear) partial differential equations (PDEs) which describe the spatio-temporal dynamics of biomass and nutrients.

#### Model simulation

The non-linear nature of the model candidates made the analytical approach impossible. Therefore, numerical techniques were employed to simulate the models. The domain of interest was discretized into a number *N* of smaller sub-domains. The use of a low order polynomial interpolation in each sub-domain allows to approximate the PDEs by a set of algebraic or ordinary differential equations (AEs or ODEs).

We considered two numerical approaches. The first combines the finite differences scheme in space - with centered differences for the nutrients and a backwards-forward space for the biomass- and the Crank-Nicolson approach in time (Balsa-Canto et al., 2017). A Newton-Raphson algorithm was used to solve the resulting non-linear equations. The second combines the finite differences scheme as implemented in MATMOL (Vande Wouwer et al., 2014) (www.matmol.org) with ode15 s (implicit ODE solver included in MATLAB). Both approaches were handled in AMIGO2 (Balsa-Canto et al., 2016) using the black-box model option. Results obtained with both implementations coincide thus confirming the reliability of the numerical methods.

#### Model identification

Model identification consisted of two steps. First, we estimated unknown model parameters for each model candidate; second, we selected the final model attending to the goodness of fit.

Parameter estimation was formulated as a non-linear optimization problem to find the unknown model parameters which minimize the distance between the model predicted values and the available data. We solved the model under the specified experimental conditions for given parameter values and results were used to obtain maximum thickness (MxT) and average nutrient (AvgN) in the bulk liquid.

We computed MxT taking into account the location of the moving boundary, the interface between the biofilm and the bulk. We computed the average nutrient (AvgN) in bulk as *mean*[*C*_*N*_(*idx*)] being *idx* the indexes that correspond to the bulk liquid.

AMIGO2 toolbox (Balsa-Canto et al., 2016) was used to solve the parameter estimation problem. In particular, we selected the least squares approach. The problem is formulated as:

Find **θ** to minimize:

(1) $$\begin{array}{l} J_{ml}=\overset{n_{s}}{\underset{i}{\sum }}{\left(mMxT_{i}-MxT_{i}\left(\theta \right)\right)}^{2}+\overset{n_{s}}{\underset{i}{\sum }}{\left(mA\upsilon gN_{i}-A\upsilon gN_{i}\left(\theta \right)\right)}^{2} \end{array}$$

subject to the dynamic constraints (the model) and bounds on the parameters **θ**_*L*_ ≤ **θ** ≤ **θ**_*U*_.

In Equation (1), *n*_*s*_ regards the number of sampling times, mMxT and mAvgN the measured MxT and AvgN; **θ** corresponds to the vector of unknown model parameters.

The global optimizer eSS (Egea et al., 2010) was used to solve the parameter estimation problem due to its well-recognized efficiency and robustness.

Input files for simulation and parameter estimation are publicly available at AMIGO2 web page: https://sites.google.com/site/amigo2toolbox/examples. Parameter ranges are included in Supplemental Data.

#### Parametric sensitivities

Local parametric sensitivities for a given observable *o* and at a sampling time *t*_*s*_ are defined as follows:

(2) $$\begin{array}{ll} S_{p}^{o}\left(t_{s}\right)=\frac{\partial y^{o}}{\partial \theta_{p}}\left(t_{s}\right); & p=1\ldots n_{\theta } \end{array}$$

where *y*^*o*^ refers to either *MxT* or *AυgN*.

The corresponding relative sensitivities, $s_{p}^{o}=\frac{△\theta_{p}}{△y^{o}}\frac{\partial y^{o}}{\partial \theta_{p}}$, can be used to asses the individual local parameter influence or importance, that is to establish a ranking of parameters.

As an overall measure of the parametric sensitivity we will use:

(3) $$\begin{array}{l} \delta_{p}^{o}=\overset{n_{s}}{\underset{s=1}{\sum }}|s_{p}^{o}\left(t_{s}\right)| \end{array}$$

## 3. Results and discussion

### Quantitative image analysis, cell counts, nutrient consumption data and modeling can be combined to investigate the dynamics of biofilm structures

**Figure 1:**
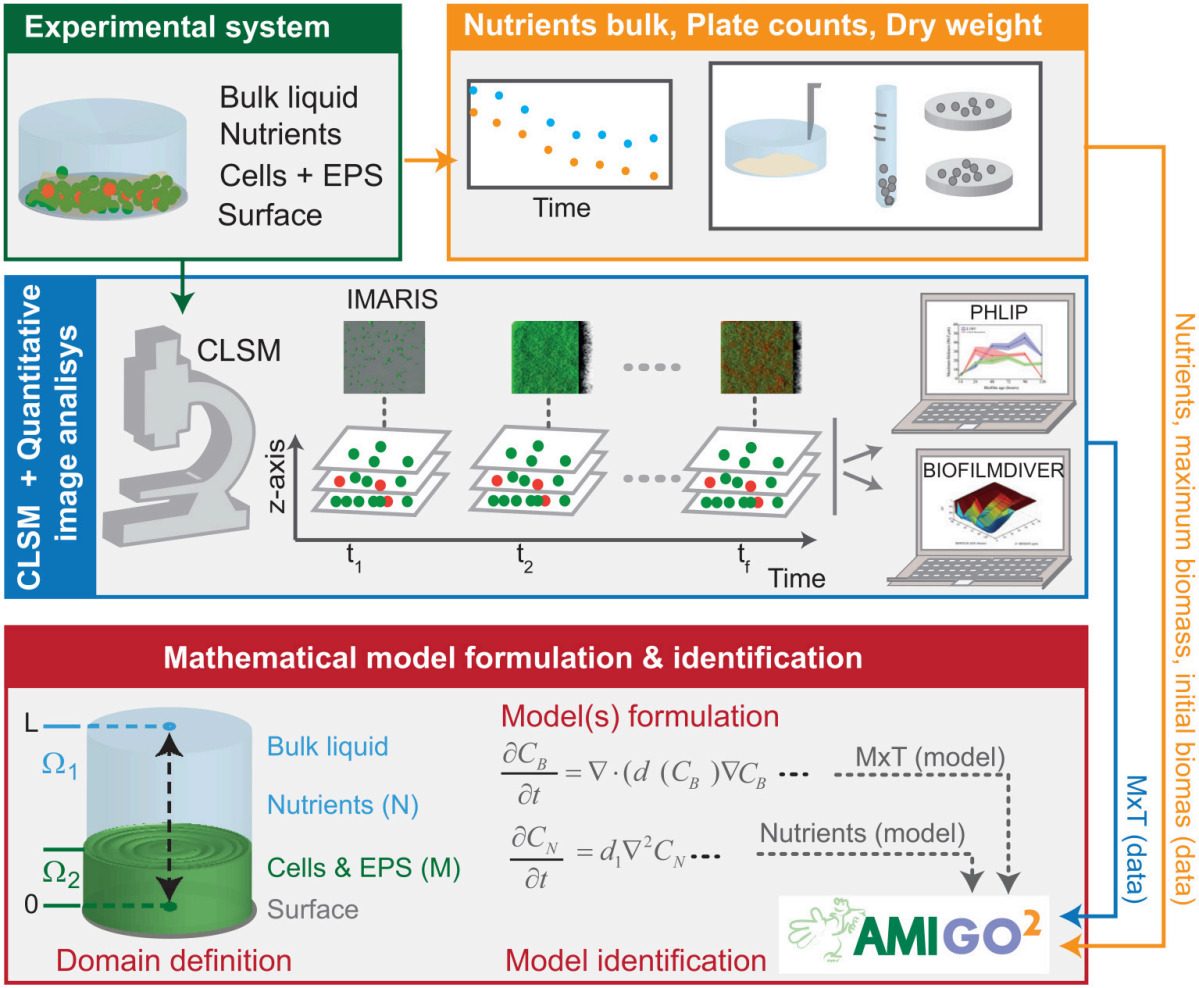
Model identification scheme based on CLSM and nutrients consumption measurements. Biofilms were grown under static conditions. CLSM was used to gather image stacks in several sampling times. IMARIS allowed reconstructing 3D-structures and quantifying maximum biofilm thickness throughout time. BIOFILMDIVER enabled computing biofilm covered area as a function of time and z-axis. Nutrients consumed by cells were measured at each sampling time. We defined candidate models, estimated unknown parameters and selected the most appropriate model using data fitting in the AMIGO2 toolbox.

The scheme in Figure 1 is intended to reconcile model candidates with experimental data through parameter estimation and model selection. The approach combines experimental data from different sources: CLSM quantitative image analysis considering two colored channels, green for viable cells and red, for damaged or dead cells; cell counts; nutrients measurements through time and modeling to test different mechanisms that drive the life cycle of bacterial biofilms.

Recent works (Mosquera-Fernández et al., 2014, 2016) suggest that biofilm maximum thickness and covered area are the most informative parameters to quantify structure dynamics. In this work we selected IMARIS to reconstruct CLSM images, PHLIP to compute thickness throughout time and BIOFILDIVER to compute covered area as a function of time and z-axis. We also measured nutrients consumption in the selected sampling times as well as the number of adhered cells.

Alternative biological hypotheses generate alternative candidate models. The most appropriate model can be then selected, attending to its capability to explain the time-resolved data by model data fitting using the AMIGO2 toolbox (Balsa-Canto et al., 2016).

### L1A1 *L. monocytogenes* forms flat, thick structures and experiments a massive detachment after 96 h

**Figure 2:**
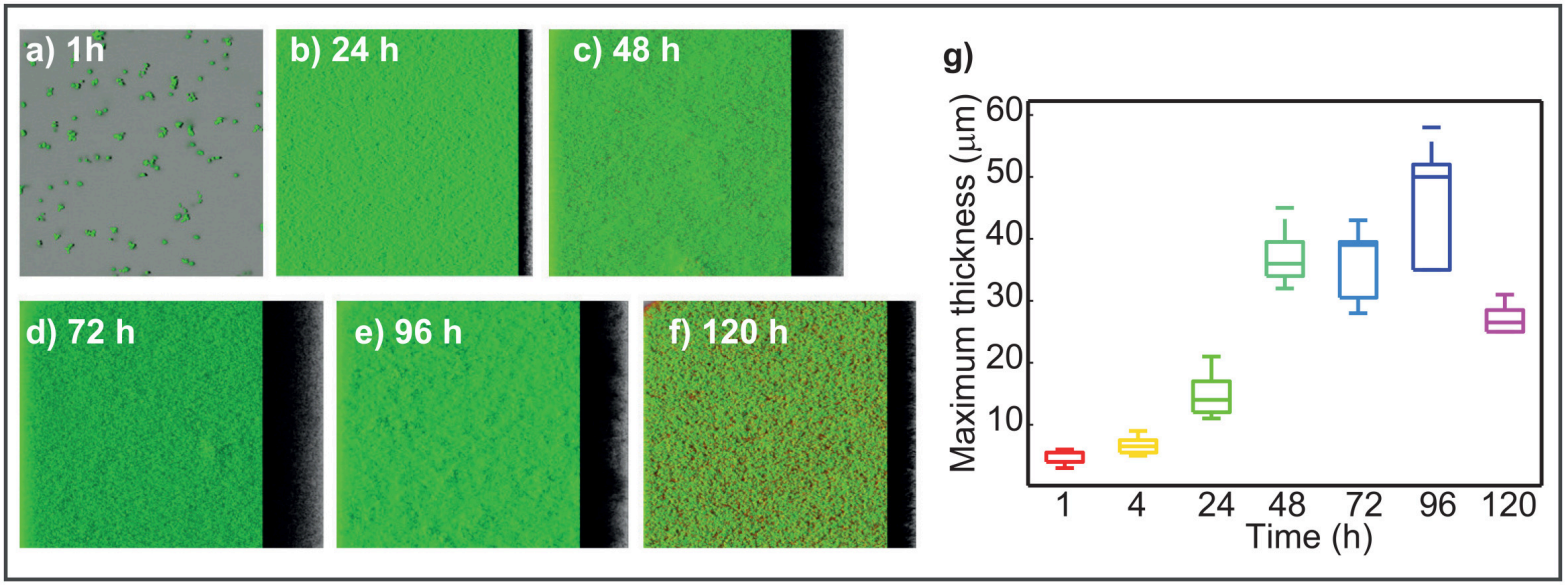
Dynamics of L1A1 *L. monocytogenes* biofilms during life cycle. **(a–f)** Present the three-dimensional reconstruction of the CLSM images captured at different times of the biofilms life cycle. **(g)** Presents the measured maximum thickness (mMxT) vs. time. Box plots represent the variability of maximum thickness over the different replicates. On each box, the central mark indicates the median, and the bottom and top edges of the box indicate the 25 and 75th percentiles, respectively. The whiskers extend to the most extreme data points not considered outliers. The figure shows the median of MxT value increases with time up to 96 h. Maximum median value is of around 50 μm. After that MxT values decrease revealing a massive detachment episode between 96 and 120 h.

CLSM three-dimensional reconstructions (Figures 2a–f) show dense and homogeneous biofilms with scattered damaged or dead cells as stained by propidium iodide. Under the experimental setup used in this study, this strain forms rather flat, unstructured biofilms. After initial attachment, a thin biofilm is already present at 24 h. The flat structure is rather stable throughout time with a sustained thickness increase until 96 h. After 96 h the presence of damaged or dead cells is high.

The measured maximum thickness (mMxT) of the biofilms was computed for each of the experimental replicates over time. The distribution of maximum thickness values is shown in the Figure 2g in the form of box plots. The boxes indicate the degree of variability and skewness in the data. Results show how the median of the maximum thickness increases with time until 96 h. The median mMxT value at this time corresponds to 50 μm.

Rieu et al. (2008) showed the differences between the MxT value achieved in static and flow conditions for the AR0009 strain. A maximum value of 10 μm was achieved in static conditions after 48 h. Bridier et al. (2010) considered the maximum thickness at 24 h of the biofilms formed by 60 pathogens including 10 *L. monocytogenes* strains. Their results reveal values ranging from 16 to 35 μm for *L. monocytogenes* strains. Guilbaud et al. (2015) computed the mean thickness achieved by 96 different strains under static flow conditions. Their results reflect that most of the strains form biofilms with a mean thickness value in the range 15-25 μm at 48 h. The fact that L1A1 biofilms were around 36 μm at 48 h indicates that this strain forms quite thick biofilms as compared to other strains.

At 120 h the MxT decreases importantly indicating a massive detachment in that period of 24 h. The maximum relative variability among experimental replicates for a specific sampling time (*t*_*s*_) can be computed as $\frac{\left[(max(MxT(t_{s}))-min(MxT(t_{s})\right]}{mean(MxT(t_{s}))}$. The maximum relative variability (19%) is found at 96 h. This may indicate that some replicates may experience a lighter detachment before 96 h. Massive detachment occurs for all replicates after 96 h.

### Covered area data reveals that live and damaged or dead cells are arranged in a multi-layer structure

**Figure 3:**
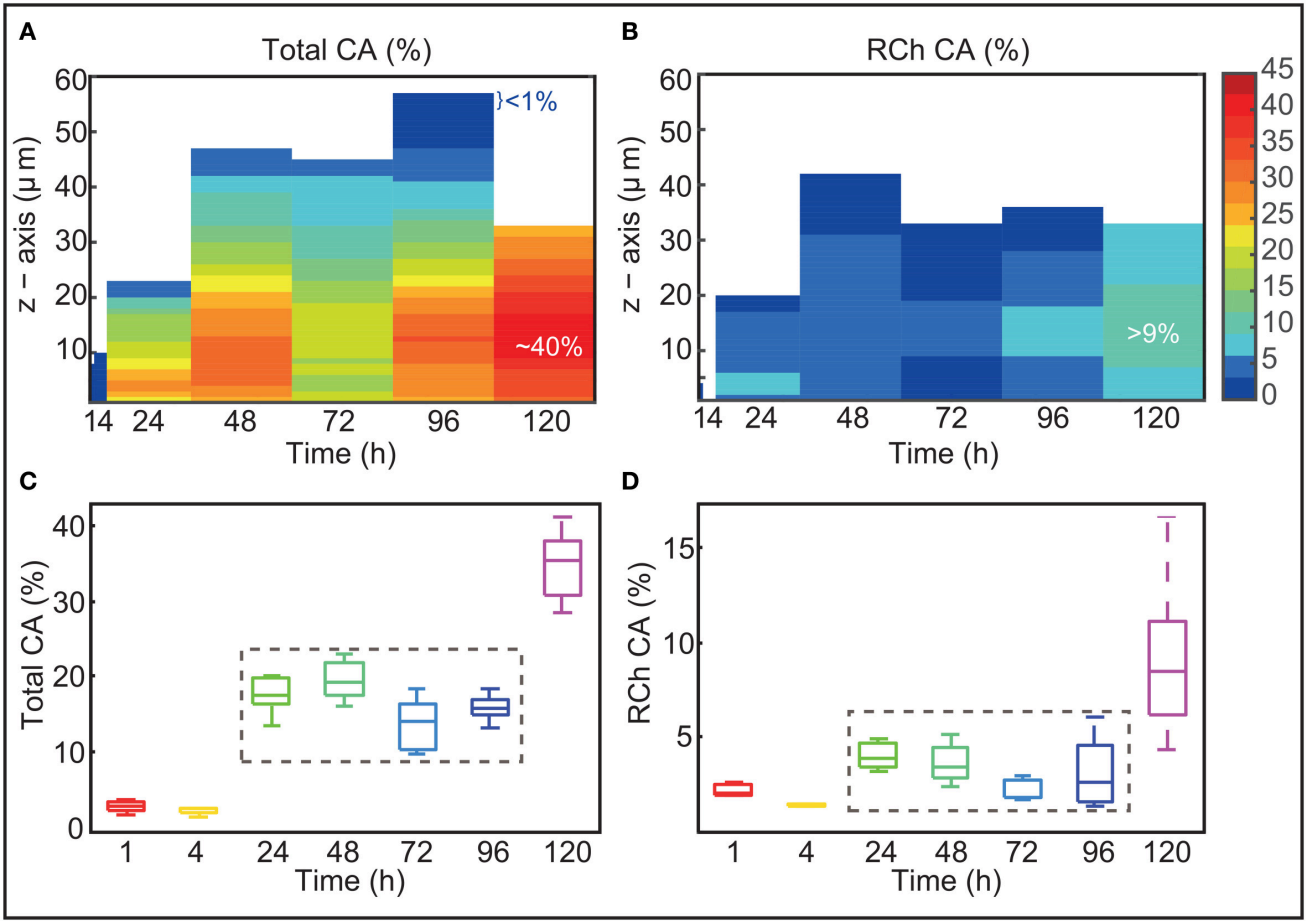
Covered area vs biofilm thickness and time. **(A,B)** Present the mean CA values obtained over experimental replicates as functions of time and thickness (*z*-axis). The Figures show that biofilms are structured in layers of different CA values. Maximum CA is around 40%. At 120 h damaged or dead cells account for almost half of the area covered by the biofilm. **(C,D)** Present the mean CA values over all slices as a function of time for the different replicates. Box plots provide information on the variability among the different replicates. CA values are kept almost constant from 24 to 96 h. At 120 h CA increases substantially.

The covered area was calculated for the different slices, channels and sampling times. Figures 3A,B present the mean values obtained over experimental replicates as functions of time and thickness (*z*-axis). The figures reflect that at each sampling time the biofilms are structured in layers with different CA values. Figure 3A presents the total covered area. The Figure shows how the maximum CA values are found in intermediate layers while CA is lower in the deepest layers and even lower toward the surface of the biofilm. Covered areas of <1% on the surface of the biofilm may reflect certain roughness of around a couple of μm or may correspond to weakly adhered cells. Lower covered areas in the deepest layers (CA ≤ 20%) might indicate the presence of voids or molecules that are not stained (including exopolysaccharides, proteins and eDNA). Figure 3B presents the results obtained for the red channel. The Figure shows the presence of damaged or dead cells from early times and in deep layers of the biofilm. Only at 120 h damaged or dead cells appear also in the surface of the biofilm.

Figures 3C,D present the evolution of the mean CA over all slices as a function of time for the different replicates. Box plots provide information on the variability among the different replicates. Figure 3C shows that mean CA is little at the earliest times. These low values indicate that at early times, cells tend to adhere on top of small clusters (of around 5 μm). Between 4 and 24 h there is a massive growth and deposition of cells which contribute to a substantial increase in biofilm dispersal. The CA reaches values in the range 15−20% which are kept until 96 h. The fact that covered area is maintained to a somehow constant value from 24 to 96 h may suggest that biofilm is only growing vertically. CA increases quite drastically from 96 to 120 h when a massive detachment occurs. This may suggest that some detached cells may be reallocated in the biofilm.

When analyzing the results obtained for the red channel (Figure 3D) we discover that a small amount of damaged or dead cells already appear at around 24 h in the deepest layers of the biofilm. The area occupied by damaged or dead cells is <6% for most of the time. Only at 120 h they appear distributed throughout the biofilm with CA values ranging between the 8% and the 12%. Remarkably at this time, damaged or dead cells account for almost half of the area covered by the biofilm, suggesting an episode of massive death which correlates with the large detachment described before.

Figures 2c,d show good reproducibility of results in most of the sampling times. A larger variability is observed in the red-channel values at 120 h. This may indicate that for the different replicates cells start dying massively at different times after 96 h thus contributing to a larger variability in the presence of damaged or dead cells.

Remarkably CA values are rather low throughout time whereas 3D-reconstructions (Figures 2b–f) show a large cellular distribution in the surface from 24 h. Damaged or dead cells also appear as early as 24 h. These results altogether may indicate the presence of small voids and macromolecules, including eDNA, supporting the hypothesis on the role of eDNA in L1A1 biofilms.

### Cells prefer glucose as carbon source but stop its consumption early in the biofilm life cycle

**Figure 4:**
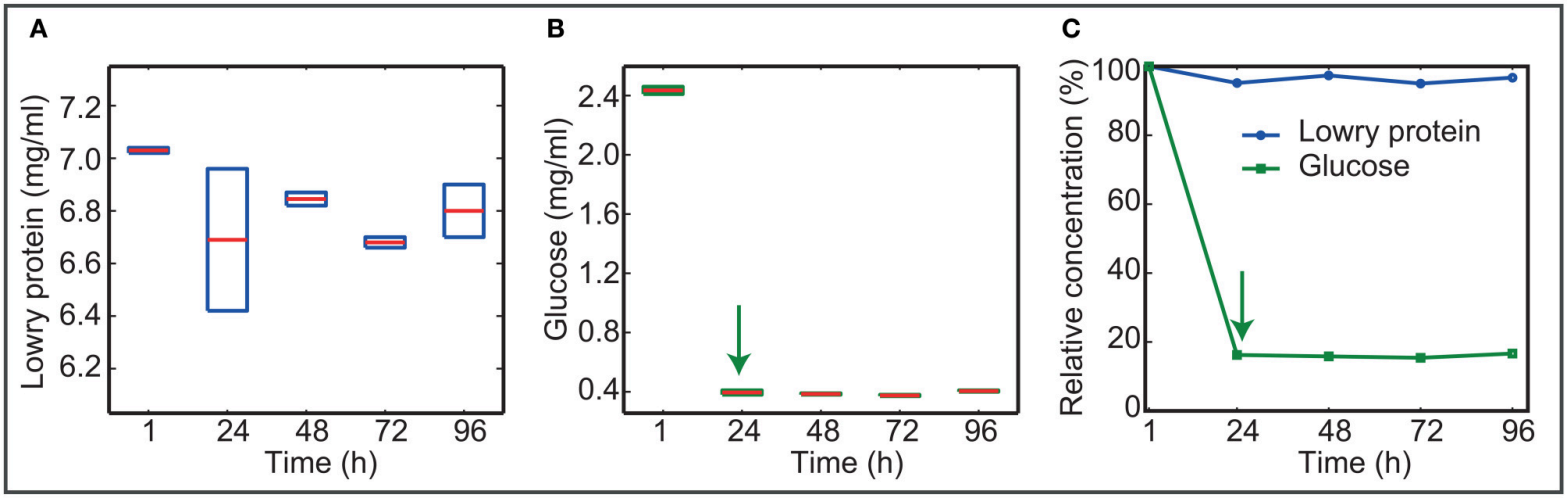
Nutrients in the bulk throughout time. **(A,B)** Present the absolute concentration of nutrients as measured in the bulk liquid. Boxplots reflect the variability among experimental replicates. **(C)** Shows the mean relative values using the concentrations at 1 h as the reference. Data show that L1A1 prefers glucose to protein as a source of nutrients. After 24 h cells stop consuming glucose.

Figure 4 presents the concentration of nutrients as measured in the bulk liquid. Data show that L1A1 consumes glucose as the primary carbon source.

Cells consume most of the glucose at early times. After 24 h glucose uptake stops even when the bulk still contains 0.4 mg/ml glucose.

### Glucose impaired uptake and biofilm aging are critical to describe the life cycle of the biofilms formed by L1A1

Biofilms were grown in static flow conditions therefore our models are restricted to the hydrostatic case. Besides, since L1A1 biofilms are rather flat, we considered a one-dimensional domain, Ω = (0, *L*), with *L* = 80 μm, in which we distinguish two time-dependent regions. Ω_1_(*t*), represents the bulk, and Ω_2_(*t*), represents the part occupied by the biomass (See Figure 1 for the graphical representation). Both regions can be characterized by the value of the normalized biomass concentration, *C*_*B*_(*t, x*) = *C*_*B*_/*B*_*max*_ and the normalized nutrients concentration, *C*_*N*_(*t, x*) = *C*_*N*_/*N*_0_. *B*_*max*_ corresponds to the maximum biomass concentration and *N*_0_ corresponds to the initial nutrients concentration, both extracted from the experimental data.

We defined four candidate models (M1-M4), from the simplest reaction-diffusion model (RDM) with constant biomass diffusion and a linear biomass detachment, to the most sophisticated model, including glucose impaired uptake and a non-linear detachment due to biofilm aging. All candidate models can be embedded in the following mathematical formulation:

(4) $$\begin{array}{l} \frac{\partial C_{N}}{\partial t}=\nabla \cdot \left(d_{N}\left(C_{N}\right)\nabla C_{N}\right)-K_{1}G_{N}\left(C_{N}C_{B}\right) \end{array}$$

(5) $$\begin{array}{l} \frac{\partial C_{B}}{\partial t}=\nabla \cdot \left(d_{B}\left(C_{B}\right)\nabla C_{B}\right)+K_{3}G_{N}\left(C_{N}C_{B}\right)-K_{4}G_{D}\left(C_{N},C_{B}\right) \end{array}$$

Equation 4 describes the dynamics of nutrients. The first term in the right-hand side accounts for the diffusion of the nutrients and the second corresponds to nutrients uptake; *d*_*N*_ is the diffusion coefficient which is different in the bulk and in the biofilm; $K_{1}=\frac{\mu_{B}}{Y_{BN}}\frac{B_{max}}{N_{0}}$ is proportional to the ratio between the maximum specific growth rate of the microorganisms (μ_*B*_) and the nutrient growth yield *K*_4_ = *Y*_*BN*_*m*_*s*_ and $K_{4}=\frac{\mu_{B}m_{s}B_{max}}{Y_{bn}}$ which is proportional to the maintenance coefficient *m*_*s*_.

Equation 5 describes the dynamics of the biofilm. In the right-hand side, the first term accounts for the diffusion of the biomass, the second for the production of biomass and the last one corresponds to the decay or detachment; *d*_*B*_ is the diffusion coefficient and *K*_3_ = μ_*B*_/*B*_*max*_. We explored several possibilities for the definition of *G*_*N*_ and *G*_*D*_.

Equations 4 and 5 are similar to the ones used in the study of mass transfer and conversion in biofilms (Picioreanu et al., 2000; Eberl et al., 2001).

In our case bacteria adhere to the surface at *x* = 0, with a given thickness of 4.5 μm and there is neither flux of bacteria nor nutrients. Mathematically, boundary and initial conditions for biomass read as follows:

(6) $$\begin{array}{l} \frac{\partial C_{B}}{\partial x}\left(t,0\right)=0,\;t\in \left[0,T\right], \end{array}$$

(7) $$\begin{array}{l} \frac{\partial C_{B}}{\partial x}\left(t,L\right)=0,\;t\in \left[0,T\right], \end{array}$$

(8) $$\begin{array}{l} C_{B}(0,x)=\{\begin{array}{ll} B_{0}, & \text{if }0\leq x\leq 4.5,\\ 0, & \text{ if }4.5<x\leq L. \end{array} \end{array}$$

Moreover, a fixed nutrients concentration is fed at *t* = 0:

(9) $$\begin{array}{l} \frac{\partial C_{N}}{\partial x}\left(t,0\right)=0,\;t\in \left[0,T\right], \end{array}$$

(10) $$\begin{array}{l} \frac{\partial C_{N}}{\partial x}\left(t,L\right)=0,\;t\in \left[0,T\right], \end{array}$$

(11) $$\begin{array}{l} C_{N}\left(0,x\right)=1,\;x\in \left[0,L\right], \end{array}$$

All candidate models present the following features:

- There is a sharp front of biomass at the bulk/solid transition. This front can be used to determine the biofilm thickness.
- Biomass density can not exceed a maximum bound *B*_*max*_ which is a parameter of the model restricted by the measured cell counts.
- Biomass production is due to nutrient consumption.
- Nutrients diffuse in the bulk and in the biofilm with different diffusion constants:
  (12) $$\begin{array}{ll} d_{N}\left(C_{N}\right)=d_{N}, & x\in \Omega_{1} \end{array}$$
  (13) $$\begin{array}{l} d_{N}\left(C_{N}\right)=d_{eff}d_{N},x\in \Omega_{2}, \end{array}$$
  where *d*_*N*_ corresponds to the constant glucose diffusivity in the bulk and *d*_*eff*_ is the effective glucose diffusivity within the biofilm (*d*_*eff*_ = 0.24; Stewart, 1998).

We estimated the unknown parameters for the different candidate models using the time resolved data of biofilm maximum thickness and average nutrient concentration in the bulk. Cell counts and cell dry weight were used to define initial conditions and bounds on the parameters. Details on the parameters to be estimated, bounds and problem formulation are included in the section Theoretical Methods (Table 1).

**Table 1:** Presents the parameters and the corresponding bounds considered for parameter estimation.

**Parameter**	**Description**	**Value**	**Model**
*N*_0_ (mg/ml)	Initial glucose concentration	2.74 (Measured)	M1-M4
*B*_*max*_ (mg/ml)	Maximum biomass	≥15.55 (Measured)	M1-M4
*d*_*N*_ (m^2^/s)	Glucose diffusivity (bulk)	1 × 10^−14^−6.6 × 10^−10^	M1-M4
*d*_*eff*_ (d)	Effective glucose diffusivity (biofilm)	0.24 (Stewart, 1998)	M1-M4
*mu*_*m*_ (1/s)	Maximum growth rate	1 × 10^−5^−5 × 10^−2^	M1-M4
*Y*_*BN*_ (d)	Biomass yield	1 × 10^−3^−1	M1-M4
*d*_*B*_ (m^2^/s)	Biomass diffusivity	1 × 10^−16^−1 × 10^−15^	M1,M3,M4
*m*_*s*_ (1/s)	Maintenance coefficient	3 × 10^−6^−5 × 10^−5^	M1-M4
ϵ	Biomass diffusivity related constant	1 × 10^−9^−1 × 10^2^	M2
*a* (d)	Biomass diffusivity related constant	0−4 (Eberl et al., 2001)	M2
*b* (d)	Biomass diffusivity related constant	0−4 (Eberl et al., 2001)	M2
*N*_*min*_ (d)	Threshold for glucose impaired uptake	0.128−0.153	M3-M4
*k*_*d*_	Rate of activation of detachment	200−400	M4
*D*_*min*_ (d)	% damaged or dead cells before detachment	0.05−0.1	M4

*Parameter bounds considered for model identification. (d) Corresponds to dimensionless parameters*.

**Figure 5:**
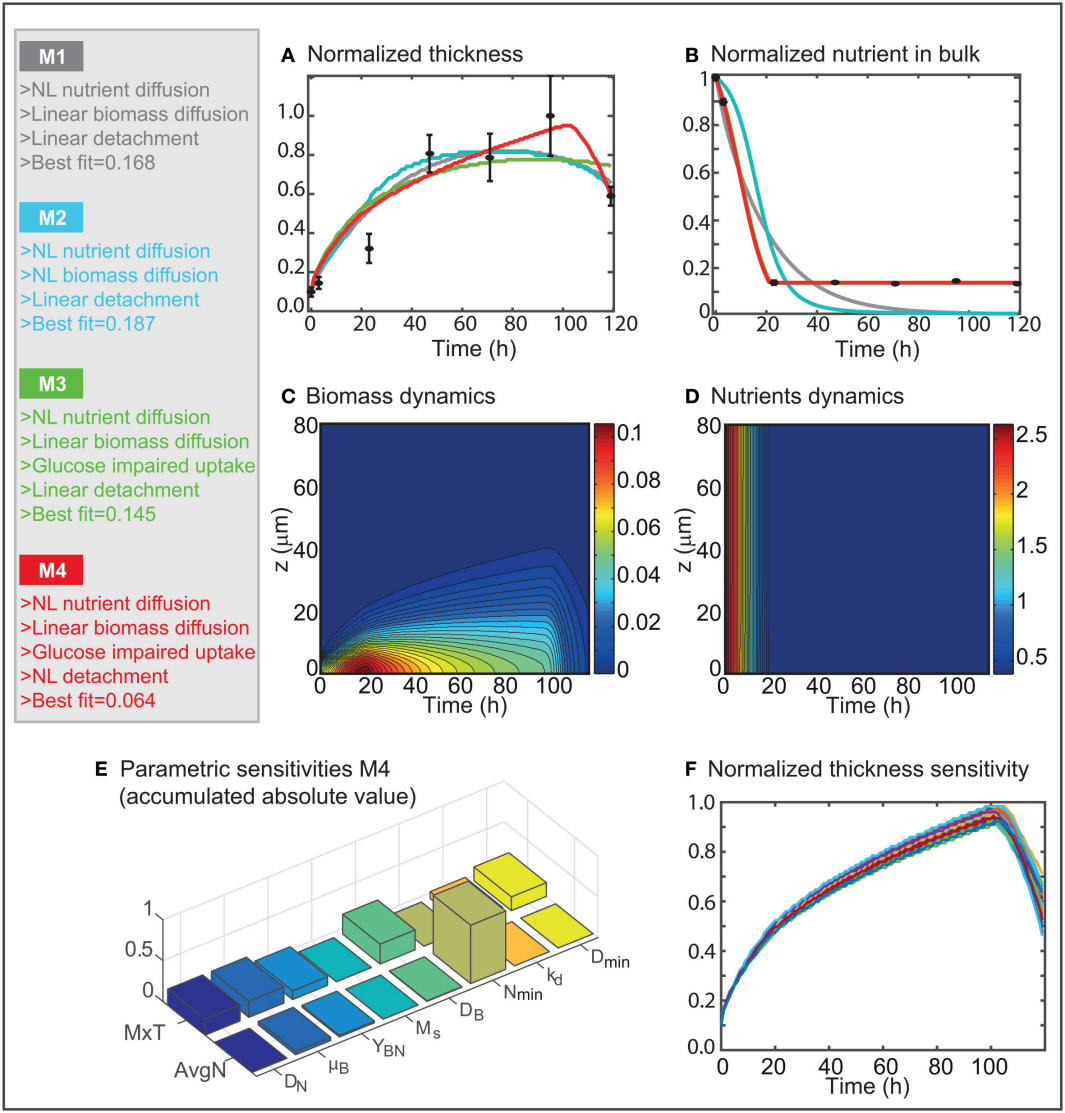
Candidate models analysis. **(A,B)** Present the best fit to the data obtained for the different candidate models. **(C,D)** Show the spatio-temporal dynamics of the biomass and nutrients concentrations as predicted by the most successful model M4. **(E)** Shows the absolute value of the relative parametric sensitivities as computed for M4. **(F)** Presents the effect of modifying the parameter values on MxT.

Figure 5 presents an overview of the candidate models and the corresponding best fit in terms of least squares error.

Model 1 (M1) assumes that biomass diffusivity is constant. Since there is an excess of nutrients in the medium, we assume that *G*_*N*_ follows a mass action description *G*_*N*_ = *C*_*N*_*C*_*B*_ instead of the usual Monod formulation. Note that we also tested the Monod formulation without improvements in the sense of goodness of fit (data not shown). Detachment is assumed to be linear and dependent on *C*_*B*_ as in, for example, Eberl et al. (2001).

Results in Figures 5A,B reveal that the standard reaction-diffusion models typically used to describe biofilms dynamics can not predict L1A1 biofilm life cycle. M1 predicts that cells consume all nutrients. Once the nutrients are exhausted the cells enter a phase of maintenance in which the thickness does not increase. Finally, the decay term dominates the growth term, and biomass depletion starts once cells maintenance is no longer possible. The maximum biofilm thickness is lower than the maximum found by quantitative image analysis, and decay starts earlier than expected.

In order to better reflect maximum thickness and the time when it is achieved, we proposed the model 2 (M2). M2 includes the hypothesis that biomass spreading is significant once a certain density value is approached. To model this scenario we selected the expression proposed by Eberl et al. (2001):

(14) $$\begin{array}{l} d_{B}\left(C_{B}\right)=C_{B}^{b}{\left(\frac{\epsilon }{B_{max}-C_{B}}\right)}^{a} \end{array}$$

where *a* and *b* are additional unknown parameters to be computed through data fitting.

The introduction of the non-linear diffusion of biomass induces a certain delay in nutrient consumption. However, the consumption becomes faster than in M1 after 20 h, thus causing thicker biofilms at early times. Both models converge to the same solution after around 60 h. The fact that the addition of two extra parameters (*a* and *b*) does not contribute to improving the quality of the fit implies that cell motility may be the driving force for cell spread at early times and not the cellular density.

Models M1 and M2 predict nutrient depletion in contrast to what it is experimentally observed. In Model 3 we hypothesize that L1A1 strain may present two glucose uptake systems with distinct affinities. Parker and Hutkins (1997) described two glucose transport systems in *L. monocytogenes* Scott A. A low-affinity proton motive force (PMF) driven system and a high-affinity phosphotransferase mediated transport system (PTS). Both systems are active at high glucose levels (early times), whereas only the PTS system is active at low glucose levels (from 24 h). This fact may be related with the expression of the virulence regulator PrfA. On the one hand, PrfA is essential for biofilm formation in *L. monocytogenes* (Lemon et al., 2010; Zhou et al., 2011) and, on the other hand, Marr et al. (2006) showed that *L. monocytogenes* strains expressing high levels of the virulence regulator PrfA show a significantly reduced expression of PTS components of the glucose uptake. Moreover, carbon catabolite repression of at least two genes of the PfrA virulence regulon previously demonstrated in *L. monocytogenes* could explain the low level of biofilm formation observed at early times of culture (Milenbachs et al., 1997).

Model 3 (M3) is equal to M1 but includes the hypothesis of two glucose transport mechanisms, in such a way that up to a certain level of glucose (*N*_*min*_), cells consume glucose following the mass action kinetics (as in M1) and once *N*_*min*_ is achieved, glucose uptake is impaired.

M3 results in a better quality of fit (Figures 5A,B) since it can recover the glucose impaired uptake. However, decay is no longer recovered, and the predicted maximum thickness is lower than the one measured.

Model 4 is intended to describe detachment. The biological, chemical, and physical factors that drive detachment are complex and not fully understood yet. Chambless and Stewart (2007) reviewed the phenomena hypothesized as factors influencing detachment. The list includes fluid shear, the degradation of the EPS, absence of sufficient nutrients or quorum sensing, to name a few. From the point of view of modeling, fluid shear and substrate limited detachment have received notable attention (Xavier et al., 2005; Chambless et al., 2006). However, these mechanisms are not suitable to describe the system under consideration in the present work. Biofilms are grown in static conditions, so that fluid shear is assumed to be negligible and nutrients are not consumed.

We hypothesize that the large detachment observed after 96 h is related to cell death and the degradation of the extracellular DNA (eDNA), i.e., to biofilm aging. The presence of eDNA in biofilms appears to be associated with both lysis of cells and active secretion (Whitchurch et al., 2002). Following cell death, a sub-population of the dead bacteria lyse and releases DNA (Bayles, 2007). Harmsen et al. (2010) showed that *L. monocytogenes* eDNA might be the only central component of the biofilm matrix. Their work suggests that eDNA has a pronounced effect on the initial attachment of cells in static assays. Also the use of DNase I treatment resulted in an extensive removal of cell material at late stages of biofilm formation essays.

In Model 4 (M4) we introduced the hypothesis of the role of aging into the detachment term. As a measure of aging, we used the measured covered area of damaged or dead cells [*CBD*(*t*)] in such a way that the decay starts once a given value of damaged or dead cells is present in the biofilm. Mathematically, this reads as follows:

(15) $$\begin{array}{l} G_{D}\left(C_{B}\right)=\frac{C_{B}}{1+exp\left(-k_{d}\left(CBD\left(t\right)-D_{min}\right)\right)} \end{array}$$

where *G*_*D*_(*C*_*B*_) is a s-shaped function centered in *D*_*min*_ and with slope *k*_*d*_.

M4 is closer to the data (Figures 5A,B). Remarkably the least squares error is half the values achieved by M1–M3. The maximum biofilm thickness is in good agreement with the experimental data throughout time. The model predicts that the highest thickness is achieved at around 102 h. After that moment cells detach massively.

Figures 5C,D present the spatio-temporal dynamics of the biomass and nutrients in the domain as obtained for model M4. Results reveal that the biomass concentration is higher in layers which are deep in the biofilm, in particular between 10 and 40 h. The fast consumption of nutrients in the first hours (1–20 h) contributes to achieving the higher biomass concentration values at early times.

Parameter values corresponding to the optimal solution are reasonable. The diffusion coefficient for dilute glucose in water at 25°C is 6.6 × 10^−10^; the glucose diffusion in the model corresponds to $d_{N}=1.07\times 10^{-11}$, which is reasonable taking into consideration that the bulk liquid contains other nutrients than glucose (e.g., proteins). The diffusivity in the biofilm is 24% that value (Stewart, 1998). To compensate for a rather low diffusivity of nutrients, the nutrient growth yield factor is rather large *Y*_*BN*_ = 0.98 which would indicate that the cells use very effectively the nutrients to grow and to produce EPS which would contribute to achieve high biomass concentrations at early times. The growth rate $\mu_{B}=8.5\times 10^{-3}$ is within the range of values published for *L. monocytogenes* species under different stress conditions (Augustin and Carlier, 2000). Note that this value does not correspond to the cellular growth but to the biofilm growth. The maintenance coefficient *Ms* = 3.66 × 10^−5^ would indicate that cells may survive up to around 7 h without access to nutrients. $N_{min}=1.39\times 10^{-1}$ corresponds to an average nutrient concentration of 0.38 which is a bit over the average nutrient value at the end of the process. $D_{min}=5.21\times 10^{-2}$ would indicate that a covered area of *CA* = 5.21% of damaged or dead cells would determine the beginning of the detachment which is rapidly linear as shown by the high value of *k*_*d*_ = 311.5.

To assess the influence of the different mechanisms into the biofilm dynamics, we computed the absolute relative parametric sensitivities at the optimum (see section Theoretical Methods). Results (Figure 5E) show that nutrients dynamics is critically affected by the glucose impairment parameter (*N*_*min*_). The biofilms growth and the yield are also influencing the nutrients consumption. The biofilm thickness is affected by all parameters. The most relevant mechanisms are the biomass diffusion and growth as well as aging. Figure 5F shows how the dynamics of the maximum thickness varies when modifying the model parameters using a normal distribution with a 5% standard deviation. The model is quite robust to parameters' modifications, the maximum thickness is consistently achieved at around 100 h presenting a low dispersion (around 10%). Furthermore, *D*_*min*_ and *k*_*d*_ have a considerable effect on the dynamics of detachment, both regarding intensity and velocity.

## 4. Conclusions

*Listeria monocytogenes* is a food-borne pathogen that can cause systemic infections in humans. In some cases food contamination has been linked to the capability of these bacteria to form biofilms, microbial structures with enhanced resistance to biocides. Therefore, it is of the highest interest to gain new insights into the life cycle of biofilms to design new elimination strategies.

These insights can be gained from a multidisciplinary approximation in which measurements and hypotheses are reconciled through a model identification procedure.

In this work, we proposed such a procedure combining quantitative CLSM image analysis, cell counts and nutrient consumption measurements with computationally efficient modeling and parameter estimation techniques.

The procedure allowed us to compare several candidate models, i.e., hypotheses, and to find the model, i.e., the mechanisms, that better describe *L1A1 L. monocytogenes* biofilms life cycle.

Remarkably glucose impaired uptake and biofilm aging are crucial to explain biofilms maximum thickness and the observed massive detachment in static cultures.

## Author contributions

EB formulated the models; CVi and AL implemented the numerical simulations; CVi and CVá designed and supervised the numerical simulations; CVi and EB performed the optimizations; MM, MC, and RB designed and performed the experiments; EB designed the work and drafted the document; all authors read and approved the manuscript.

### Conflict of interest statement

The authors declare that the research was conducted in the absence of any commercial or financial relationships that could be construed as a potential conflict of interest.
