# Supplementary material for: Modeling Reveals the Role of Aging and Glucose Uptake Impairment in L1A1 Listeria monocytogenes Biofilm Life Cycle
Source: Front Microbiol. 2017 Nov 1;8:2118. doi: 10.3389/fmicb.2017.02118 (PMC5671982; doi:10.3389/fmicb.2017.02118)
Supplement: Supplementary file 1 [file Table1.pdf]

## SUPPLEMENTAL DATA

Table 1 presents the parameters and the corresponding bounds considered for parameter estimation.

| Parameter | Description | Value | Model |
|---|---|---|---|
| $N_0$ $(mg/ml)$ | Initial glucose concentration | 2.74 (Measured) | M1-M4 |
| $B_{max}$ $(mg/ml)$ | Maximum biomass | $\geq$ 15.55 (Measured) | M1-M4 |
| $d_N$ $(m^2/s)$ | Glucose diffusivity (bulk) | $1 \times 10^{-14} - 6.6 \times 10^{-10}$ | M1-M4 |
| $d_{eff}$ (d) | Effective glucose diffusivity (biofilm) | 0.24(Stewart, 1998) | M1-M4 |
| $mu_m$ $(1/s)$ | Maximum growth rate | $1 \times 10^{-5} - 5 \times 10^{-2}$ | M1-M4 |
| $Y_{BN}$ (d) | Biomass yield | $1 \times 10^{-3} - 1$ | M1-M4 |
| $d_B$ $(m^2/s)$ | Biomass diffusivity | $1 \times 10^{-16} - 1 \times 10^{-15}$ | M1,M3,M4 |
| $m_s$ $(1/s)$ | Maintenance coefficient | $3 \times 10^{-6} - 5 \times 10^{-5}$ | M1-M4 |
| $\epsilon$ | Biomass diffusivity related constant | $1 \times 10^{-9} - 1 \times 10^{2}$ | M2 |
| $a$ (d) | Biomass diffusivity related constant | $0 - 4$ (Eberl et al., 2001) | M2 |
| $b$ (d) | Biomass diffusivity related constant | $0 - 4$ (Eberl et al., 2001) | M2 |
| $N_{min}$ (d) | Threshold for glucose impaired uptake | $0.128 - 0.153$* | M3-M4 |
| $k_d$ | Rate of activation of detachment | $200 - 400$ | M4 |
| $D_{min}$ (d) | $\%$ damaged or dead cells before detachment | $0.05 - 0.1$ | M4 |

**Table 1.** Parameter bounds considered for model identification. (d) Corresponds to dimensionless parameters.

## REFERENCES

Eberl, H. J., Parker, D. F., and van Loosdrecht, M. C. M. (2001). A new deterministic spatio temporal continuum model for biofilm development. *J. Theor. Med.* 3(3), 161–175

Stewart, P. S. (1998). A review of experimental measurements of effective diffusive permeabilities and effective diffusion coefficients in biofilms. *Biotech. & Bioeng.* 59(3), 261–272
